# Emergence and Spread of Extensively and Totally Drug-Resistant Tuberculosis, South Africa

**DOI:** 10.3201//EID1903.120246

**Published:** 2013-03

**Authors:** Marisa Klopper, Robin Mark Warren, Cindy Hayes, Nicolaas Claudius Gey van Pittius, Elizabeth Maria Streicher, Borna Müller, Frederick Adriaan Sirgel, Mamisa Chabula-Nxiweni, Ebrahim Hoosain, Gerrit Coetzee, Paul David van Helden, Thomas Calldo Victor, André Phillip Trollip

**Affiliations:** Author affiliations: Stellenbosch University, Cape Town, South Africa (M. Klopper, R.M. Warren, N.C. Gey van Pittius, E.M. Streicher, B. Müller, F.A. Sirgel, P.D. van Helden, T.C. Victor, AP.. Trollip);; National Health Laboratory Services, Port Elizabeth, South Africa (C. Hayes, G. Coetzee); Swiss Tropical and Public Health Institute, Basel, Switzerland (B. Müller);; University of Basel, Basel (B. Müller); Nelson Mandela Metropolitan Municipality, Port Elizabeth (M. Chabula-Nxiweni);; Human Sciences Research Council, Port Elizabeth (E. Hoosain)

**Keywords:** Tuberculosis, TB, MDR TB, XDR-TB, extensively drug-resistant tuberculosis, totally drug resistant TB, South Africa, mycobacteria, *Mycobacterium tuberculosis*, bacteria

## Abstract

Factors driving the increase in drug-resistant tuberculosis (TB) in the Eastern Cape Province, South Africa, are not understood. A convenience sample of 309 drug-susceptible and 342 multidrug-resistant (MDR) TB isolates, collected July 2008–July 2009, were characterized by spoligotyping, DNA fingerprinting, insertion site mapping, and targeted DNA sequencing. Analysis of molecular-based data showed diverse genetic backgrounds among drug-sensitive and MDR TB sensu stricto isolates in contrast to restricted genetic backgrounds among pre–extensively drug-resistant (pre-XDR) TB and XDR TB isolates. Second-line drug resistance was significantly associated with the atypical Beijing genotype. DNA fingerprinting and sequencing demonstrated that the pre-XDR and XDR atypical Beijing isolates evolved from a common progenitor; 85% and 92%, respectively, were clustered, indicating transmission. Ninety-three percent of atypical XDR Beijing isolates had mutations that confer resistance to 10 anti-TB drugs, and some isolates also were resistant to *para*-aminosalicylic acid. These findings suggest the emergence of totally drug-resistant TB.

The emergence of drug-resistant tuberculosis (TB) is of major concern to TB control in South Africa. A countrywide survey in 2002 revealed that 1.8% of all new TB patients and 6.7% of TB patients who had undergone previous treatment had multidrug-resistant (MDR) TB (resistant to at least isoniazid and rifampin) (*1*). This finding translates to an estimated annual case load of 13,000 MDR TB cases, placing South Africa fourth among countries where MDR TB is highly prevalent (*1*). However, this number may be an underestimation; 2 recent studies (*2*,*3*) suggested that the proportion of MDR TB cases may be substantially higher than the World Health Organization (WHO) estimate (*3*). In addition, only 4,143 of the 9,070 patients (46%) who received a diagnosis of MDR TB in 2009 received treatment, possibly because of resource constraints, creating a situation in which control was bound to fail (*4*). This conclusion is supported by the diagnosis of 594 extensively drug- resistant (XDR) TB cases (MDR plus additional resistance to a fluoroquinolone and any second-line injectable drug) in that year (*4*). The cure rate of patients with drug-resistant TB is <50% for those with MDR TB (*5*), whereas culture conversion was observed in only 19% of XDR TB case-patients during the follow-up period, irrespective of HIV status (*6*).

Most cases of MDR TB and XDR TB in South Africa have been detected in KwaZulu-Natal, Western Cape, and Eastern Cape Provinces (*4*). Statistics from the Eastern Cape showed the largest increase in the number of MDR TB cases, rising from 836 cases in 2006 to 1,858 cases in 2009 (2.2 fold increase) (*4*). The reason for this dramatic increase in MDR TB cases remains to be determined.

Molecular epidemiologic data from the neighboring Western Cape Province have demonstrated that MDR TB is spread by primary transmission (*7*–*9*), which accounts for nearly 80% of reported MDR TB cases (*2*). To date, only 1 molecular epidemiologic study has been reported for the Eastern Cape (*10*), and it showed that 50% of rifampin-resistant TB isolates (including MDR TB isolates) belonged to the Beijing genotype and that “atypical” Beijing strains were significantly overrepresented. These strains harbored rare mutations in the *inhA* gene promoter (G-17A) and *rpoB* gene (GAC→GTC nucleotide substitutions in codon 516), which have previously been associated with a high fitness cost (*11*). The authors demonstrated that the spread of these strains was facilitated by HIV co-infection, thereby raising concern for the spread of drug-resistant strains in vulnerable populations (*10*).

A recent epidemiologic study conducted in the Eastern Cape estimated that 75.6% of XDR TB cases with complete data were a result of ongoing transmission (*12*). Treatment outcomes were dismal; 58% of case-patients died within 1 year, and culture conversion was observed in only 8.4% of case-patients after 143 days of treatment (*12*), raising concern that these patients had an untreatable form of TB. This situation is similar to the Tugela Ferry outbreak in KwaZulu-Natal Province (*13*), which highlighted the need for improved basic control measures, including rapid diagnostics and infection control methods (*14*).

This study aimed to describe the *Mycobacterium tuberculosis* strain population structure among MDR TB and XDR TB case-patients in Eastern Cape Province, South Africa, in order to determine whether the epidemic was driven by acquisition or transmission of resistance and to describe the extent of resistance within these strains. These findings will inform TB control efforts to better implement measures to curb emergence or the spread of drug-resistance.

## Materials and Methods

### Study Population

Sputum specimens were collected from persons at high-risk for suspected TB (previously treated case-patients and close contacts of known patients with drug- resistant cases) in accordance with the National TB Control Program. Specimens that were collected at healthcare facilities in the Eastern Cape Province were submitted to the National Health Laboratory Service (NHLS) in Port Elizabeth for TB drug susceptibility testing (DST). From July 2008 through July 2009, a convenience sample of sputum cultures, shown to be either fully drug-susceptible or resistant to at least isoniazid and rifampin (MDR TB) by the NHLS, was submitted to Stellenbosch University in Cape Town for subsequent genotyping. Only limited demographic and clinical data were available for each patient: a unique identifier (assigned by the NHLS), the date sputum was obtained, the name of the clinic/hospital where the sample originated, and the routine DST pattern. The unique identifier was used to identify the first available isolate from 309 drug-susceptible and 342 MDR TB case-patients included in the study. This study was approved by the ethics committee of Stellenbosch University, Faculty of Health Sciences (N09/11/296).

### Drug Susceptibility Testing

Sputum samples were processed by the NHLS for routine TB diagnosis by smear microscopy and culture. Each sputum specimen was decontaminated by using the standard *N*-acetyl-L-cysteine-sodium hydroxide method and cultured in mycobacteria growth indicator tube (MGIT) 960 medium until a positive growth index was observed. DST was done by the indirect proportion method with the BACTEC MGIT 960 system (BD Bioscience, Sparks, MD, USA), according to the manufacturer’s instructions. We initially tested resistance against isoniazid and rifampin, followed by testing for resistance against streptomycin and ethambutol if the isolate was resistant to either isoniazid or rifampin. Second-line DST was done in 7H10 medium containing 2 µg/mL of ofloxacin, 4 µg/mL of amikacin, or 5 µg/mL of ethionamide. DST for *para*-aminosalicylic acid was done at Stellenbosch University in MGIT 960 medium containing 4.0 µg/mL, 8 µg/mL, and 16 µg/mL of *para*-aminosalicylic acid (*15*).

### Molecular-based Analysis

Crude DNA was prepared by boiling a 200-µL aliquot of a mycobacteria-positive MGIT culture, and this was used as a template for subsequent PCR analysis (*16*). Each isolate was spoligotyped by using the international standardized method (*17*) and grouped into genotypes according to previously described spoligotype signatures (*18*). Beijing genotype strains were subclassified as either “typical” or “atypical,” according to the presence or absence of an IS*6110* insertion in the noise transfer function (NTF) region (*19*,*20*). The atypical Beijing genotype strains were further classified by using the international standardized IS*6110* DNA fingerprinting method (*21*). For atypical Beijing strains that were drug-sensitive according to DST, sensitivity to isoniazid and rifampin was confirmed by sequencing the *katG* and *rpoB* genes. In MDR atypical Beijing strains, mutations conferring resistance to isoniazid, rifampin, ethambutol, pyrazinamide, ofloxacin, streptomycin, amikacin, kanamycin, and capreomycin were identified by sequencing the *inhA* promoter and the *katG, rpoB, embB*, *pncA, gyrA*, and *rrs* genes, respectively (*22*,*23*). Isolates were grouped as as follows: MDR TB sensu stricto (MDR TB ss, that is, MDR strains excluding identified pre-XDR, MDR plus additional resistance to either a fluoroquinolone or any second-line injectable anti-TB drug) [*24*] and XDR strains); pre-XDR TB; or XDR TB, according to high confidence mutations. This method of grouping was selected because routine DST was not done for all of the anti-TB drugs on all of the isolates. Furthermore, a poor correlation was observed between high-confidence mutations and routine second-line DST. Isolates were considered to belong to the same cluster (implying ongoing transmission) if identical mutations were observed in all of the genes sequenced.

## Results

A convenience sample of 309 drug-sensitive and 342 MDR TB isolates from patients from Eastern Cape Province was collected during the study period. These isolates were submitted to Stellenbosch University for molecular-based analysis. Analysis of the population structure of these isolates by spoligotyping identified 52 and 29 different spoligotype patterns among drug-sensitive and MDR TB strains, respectively. Among drug-sensitive and MDR isolates, 22/52 and 14/29 spoligotype patterns were previously recorded in the fourth international spoligotyping (SpolDB4) database. These represented 275 (89.0%) of 309 drug-sensitive isolates and 327 (95.6%) of 342 MDR isolates. Notably, 84% of MDR isolates constituted only 3 different spoligotypes (Table 1), namely, Beijing, LAM3, and LAM4. These findings indicate transmission of these strains.

**Table 1:** Spoligotype classification of drug-sensitive and MDR TB isolates, Eastern Cape Province, South Africa, 2008–2009*

Spoligotype family†	ST no.	Culture-based DST, no. (%)					Molecular-based DST, no. (%)		
		Sensitive	MDR*ss*	Pre-XDR	XDR		MDRss	Pre-XDR	XDR
Atypical Beijing	1	11 (3.6)	41 (27.0)	98 (92.5)	78 (92.9)		29 (22.5)	85 (87.6)	103 (95.4)
Typical Beijing	1	108 (35.0)	19 (12.5)	0	0		18 (14.0)	1 (1.0)	0
H	36; 47; 50; 62	7 (2.3)	2 (1.3)	1 (0.9)	0		2 (1.6)	1 (1.0)	0
LAM3	33; 130; 211	66 (21.4)	12 (7.9)	2 (1.9)	0		12 (9.3)	0 (0)	0
LAM4	60; 811	6 (1.9)	32 (21.1)	4 (3.8)	2 (1.9)		29 (22.5)	3 (3.1)	2 (1.9)
LAM (other)	4; 20; 42; 398	7 (2.3)	1 (0.7)	0	1 (1.2)		1 (0.8)	0	1 (0.9)
MANU2	1247	0	0	0	2 (2.4)		1 (0.8)	0	1 (0.9)
S	34; 71	8 (2.6)	8 (5.3)	0	1 (1.2)		8 (6.2)	0	1 (0.9)
T	44; 53; 73; 254; 926; 1240	51 (16.5)	18 (11.8)	0	0		13 (10.1)	5 (5.2)	0
U	443; 519; 790	1 (0.3)	2 (1.3)	0	0		2 (1.6)	0	0
X	18; 92; 119; 1751	6 (1.9)	3 (2.0)	0	0		3 (2.3)	0	0
CAS	21; 26; 1092	4 (1.3)	0	0	0		0	0	0
Orphan	Not assigned	34 (11.0)	14 (9.2)	1 (0.9)	0		11 (8.5)	2 (2.1)	0
Total		309	152	106	84		129	97	108
Total MDR				342				334‡	

*MDR TB, multidrug-resistant tuberculosis; ST, shared type (*17*); DST, drug susceptibility testing; MDR*ss*, MDR sensu stricto; Pre-XDR, pre–extensively drug resistant; XDR, extensively drug resistant. †For Beijing isolates a distinction was made between typical and atypical based on the presence or absence of an IS*6110* insertion in the noise transfer region (*18**,**19*). ‡Molecular-based DST total differs from culture-based DST total, because some results were not available.

Table 1 shows the classification of spoligotypes, according to the degree of drug resistance, in which drug resistance is expressed as the result of culture-based or molecular-based DST. In this study, we used molecular-based DST to define the extent of drug-resistance in routinely diagnosed MDR TB isolates. Accordingly, 119 (38.5%) of the drug-susceptible isolates and 236 (69.0%) of the MDR TB isolates were of the Beijing genotype strain. Subclassification of Beijing genotype strains showed that 11(9.2%) of 119 drug-sensitive and 217 (91.9%) of 236 MDR strains belonged to the “atypical” subgroup of the Beijing genotype, as indicated by the absence of an IS*6110* element in the NTF region.

Analysis of mutations conferring resistance to first- and second-line anti-TB drugs enabled grouping of the MDR isolates: 136 MDR ss*,* 98 pre-XDR, and 108 XDR. Using these groupings, we found that isolates with a higher degree of resistance were more likely to have an atypical Beijing genotype (drug sensitive: 11/309 [3.6%, 95% CI 1.8%–6.3%], MDR ss: 29/136 [21.3%, 95% CI 14.8%–29.2%] vs. pre-XDR: 85/98 [86.7%, 95% CI 78.4%–92.7%] vs. XDR: 103/108 [95.4%, 95% CI 89.5%–98.5%]).

We analyzed DNA sequencing data for the first available isolate from each patient infected with an MDR atypical Beijing strain (n = 217) and performed IS*6110* fingerprinting for a subset of these isolates (n = 110) to establish whether the overabundance of the atypical Beijing genotype among patients with pre-XDR TB and XDR TB strains reflected ongoing transmission. IS*6110* DNA fingerprinting showed that all of these patients were infected with closely related atypical Beijing strains with only minor differences in the banding patterns (Technical Appendix, Figures 1, 2), thereby suggesting clonal dissemination.

The Technical Appendix Table shows that 216 (99.5%) of 217 of the MDR atypical Beijing genotype strains harbored an identical *katG* (AGC315ACC) mutation, whereas 209 (94.9%) of 217 had a distinctive *rrs* (A513C) gene mutation. This finding suggests that these mutations were acquired before dissemination. Subsequently, resistance to rifampin, ethambutol, pyrazinamide, amikacin, and ofloxacin was acquired in various combinations. Of the 29 atypical Beijing MDR ss isolates, 22 (75.9%) were grouped into 4 clusters according to mutations (mutation pattern [MP]) in the *inhA* promoter and the *katG*, *rpoB*, *embB*, *pncA, rrs,* and *gyrA* genes (cluster size ranged from 3 to 12 cases; Table 2: MP2, MP17, MP32, MP34), whereas 7 had unique MPs (Table 2: MP23, MP25, MP30, MP31, MP41, MP44, MP48). Similarly, the 85 atypical pre-XDR Beijing isolates showed 11 different MPs, of which 81 (95.3%) were grouped into 7 clusters (cluster size ranged from 2 to 62 cases; Technical Appendix Table: MP3, MP5, MP18, MP26, MP28, MP35, MP38). The genotype of the largest pre-XDR TB cluster was characterized by an *inhA* promoter mutation at position 17 and the *katG* AGC315ACC, *rpoB* GAC516GTC, *embB* ATG306ATA, *rrs* A513C, and *rrs* A1401G nucleotide substitutions as well as an insertion in the *pncA* gene at position 172G. This MP was characteristic of 81 (78.6%) of 103 of the atypical Beijing XDR TB isolates and, for ease of reference, will be called MP5 (Technical Appendix Table). By contrast, only 3 of the 29 atypical Beijing MDR ss isolates showed the same mutation pattern for these genes, excluding the *rrs*A1401G mutation (MP2). Ten different atypical XDR Beijing MPs emerged from the MP5 progenitor by mutation in the *gyrA* gene. Of these, 6 MPs showed clustering (cluster size ranged from 2 to 46 cases, MP6–11), and 4 had unique mutations conferring ofloxacin resistance (MP12–16). Clustering of both the pre-XDR and XDR genotypes suggests transmission after the acquisition of additional resistance. Of the remaining 22 atypical XDR Beijing isolates, 12 distinct resistance MPs were observed, of which 11 isolates were clustered (MP27) and 11 had unique genotypes (MP19–22, MP24, MP29, MP39–40, MP42–43, MP47).

**Table 2:** Correlation of culture-based and molecular-based drug-susceptibility testing among atypical Beijing isolates, South Africa, 2008–2009*

Drug/gene	CB-DST R, MB-DST R	CB-DST S, MB-DST S	CB-DST R, MB-DST S	CB-DST S, MB-DST R	Total	Correlation, %
INH/*katG*	217	9	1	0	227	99.6
RIF/*rpoB*	219	9	0	0	228	100
STR/*rrs*500	191	6	2	16	215	91.6
EMB/*embB*	56	5	2	152	215	28.4
ETH/*inhA* promoter	76	25	5	86	192	52.6
OFL/*gyrA*	78	93	0	29	200	85.5
AMK/*rrs*1400	167	32	7	9	215	92.6
CAP/*rrs*1400	21	38	1	155	215	27.4

*CB-DST, culture-based drug susceptibility testing; R, resistant; MB-DST, molecular-based DST; S, sensitive; INH, isoniazid; RIF, rifampin; STR, streptomycin; EMB, ethambutol; ETH, ethionamide; OFL, ofloxacin; AMK, amikacin; CAP, capreomycin.

Spatial analysis of the patients’ origins showed that pre-XDR and XDR isolates with an atypical Beijing genotype were found in 5 of 8 district municipalities (Figure; Technical Appendix Table). The largest atypical pre-XDR Beijing genotype cluster (MP5) was identified in 4 adjacent district municipalities (Technical Appendix Table), and the largest XDR TB cluster (MP6) was identified in 3 of these districts as well as in an additional district, which suggests the past spread of these genotypes.

**Figure:**
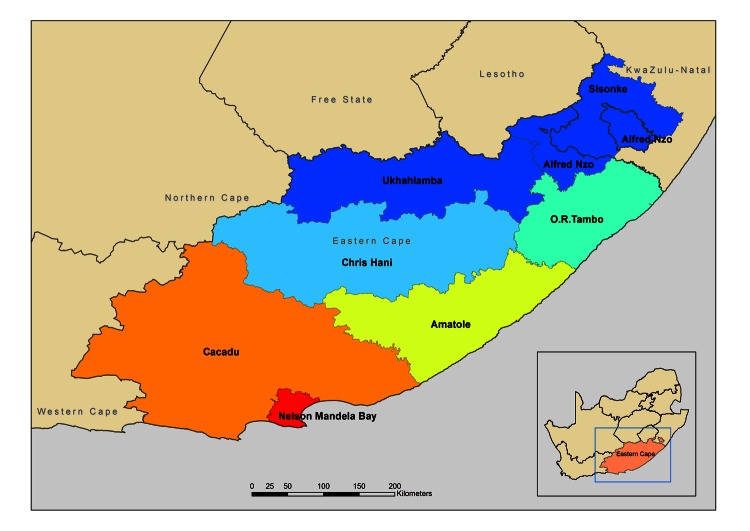
District municipalities in the Eastern Cape Province, South Africa. Map courtesy of F.W. van Zyl.

The presence of mutations in target genes known to confer resistance with high confidence indicated that 95.1% (98/103) of the atypical XDR Beijing isolates were resistant to at least 10 anti-TB drugs: isoniazid, rifampin, ethambutol, pyrazinamide, streptomycin, amikacin, kanamycin, capreomycin, ethionamide, and ofloxacin. The extent of drug resistance in these isolates was underestimated by routine DST (Table 2). The correlation between molecular-based drug-resistance and routine culture-based DST was 99.6% for isoniazid, 100% for rifampin, 28% for ethambutol, 92% for streptomycin, 93% for amikacin, 27% for capreomycin, 52% for ethionamide, and 86% for ofloxacin (Table 2). Routine DST for pyrazinamide, kanamycin, cycloserine, and *para*-aminosalicylic acid was not performed. DST for *para*-aminosalicylic acid was done at Stellenbosch University on 45 isolates; 9 showed resistance at a level of >4.0 µg/mL.

## Discussion

Review of routine DST results highlights the severity of the drug-resistant TB epidemic in South Africa (*4*) and thereby emphasizes the urgent need for curbing the rising incidence of drug resistance in the country. This result can only be achieved by implementing appropriate intervention strategies based on knowledge of the mechanisms fueling this epidemic. Recently, molecular epidemiologic techniques were used in combination with classical epidemiologic data to enhance understanding of the TB epidemic in different settings. Those studies have quantified the relative proportion of acquisition versus transmission and have described the population structure of *M. tuberculosis* over time (*7*,*10*,*22*,*24*,*25*). Using these approaches, we show that patients with MDR TB in the Eastern Cape could be divided into 2 distinct groups: isolates from patients infected with MDR ss showed diverse genetic backgrounds, while isolates from patients infected with pre-XDR TB and XDR TB showed restricted genetic backgrounds.

The finding that the pre-XDR TB and XDR TB strains are genetically distinct when compared to the MDR ss strains is counterintuitive because we would expect all MDR TB strains to have had an equal chance of acquiring resistance to second-line anti-TB drugs. The absence of second-line resistance among a large number of different MDR TB genotypes suggests that under the current MDR TB treatment regimen, acquisition of additional resistance in MDR ss strains is reduced. Conversely, analysis of the DNA sequencing data showed a significant association between the atypical Beijing genotype and mutations conferring second-line resistance. This demonstrates that this genotype has acquired resistance to the level of pre-XDR TB, which in turn has spread and thereafter has acquired additional resistance to the level of XDR TB, followed again by transmission. An alternative explanation would be that the atypical Beijing genotype acquires resistance by conferring mutations more readily than other genotypes. However, the convergent evolution of 7 different mutations within a single genotype is highly unlikely.

Analysis of the locations of the pre-XDR TB case-patients infected with this clone shows that it had a wide geographic distribution, which suggests that this genotype has been in circulation for an extended period. This conclusion was further supported by the analysis of the evolutionary order in which resistance was acquired (Technical Appendix Table), which showed that the ancestral clone first acquired resistance to isoniazid and streptomycin. This could be explained by the treatment regimen used in the early 1960s, which was based on the combination of isoniazid and streptomycin (*26*). A similar conclusion was drawn from whole genome sequence data which predicted that mutations conferring resistance to isoniazid and streptomycin were deeply rooted in the atypical Beijing genotype (*27*).

Given the extent of resistance in pre-XDR TB strains and the extremely limited treatment options available, the emergence of ofloxacin resistance was inevitable. This idea was supported by our molecular-based analysis of the XDR TB isolates, which demonstrated that resistance to a fluoroquinolone had been acquired independently on several different occasions (several different *gyrA* mutations were observed), followed by amplification through transmission (clustering of XDR phenotypes was observed). However, the true extent of acquisition may be higher than predicted, given that the XDR TB isolates were cultured from samples from patients who resided in different district municipalities, and contact was unlikely because of the long distances.

We suggest that the absence of routine second-line drug susceptibility testing and the treatment of MDR TB with an inadequate standardized regimen, according to the 2004 guidelines (www.sahealthinfo.org/tb/mdrtbguidelines.pdf) (6 months’ intensive phase: kanamycin, ethionamide, pyrazinamide, ofloxacin, and cycloserine or ethambutol; 12–18 months continuation phase: ethionamide, ofloxacin, and cycloserine or ethambutol) (*28*) may have led to the inappropriate treatment of undiagnosed pre-XDR TB cases. This regimen would have prolonged the period of infectiousness leading to transmission to close contacts and increased the risk of amplification of resistance (*28*,*29*). This problem has been recently addressed with the implementation of a revised treatment regimen (*28*) as well as routine second-line DST, which is now done on all isolates shown to be resistant to rifampin. However, these tests are culture-based, which exacerbates diagnostic delay and possible transmission. This situation can be partially resolved with the implementation of a genetic-based second-line drug susceptibility test (*29*). However, the extent of resistance associated with the atypical Beijing genotype makes treatment options extremely difficult as these isolates are resistant to all first-line anti-TB drugs (isoniazid, rifampin, ethambutol, pyrazinamide and streptomycin) and many of the second-line drugs (amikacin, kanamycin, ofloxacin, ethionamide, capreomycin). A limited number of isolates were also resistant to *para*-aminosalicylic acid. This suggests that the atypical Beijing genotype clone is evolving toward total drug resistance (defined as in vitro resistance to all first-line drugs, as well as aminoglycosides, cyclic polypeptides, fluoroquinolones, thioamides, serine analogs, and salicylic acid derivatives [*30*]) with acknowledgment of WHO’s concern over the definition (*31*). Our molecular-based results are in accordance with a recent study from the Eastern Cape, which documented extremely poor treatment outcomes for XDR TB case-patients (*12*). The authors found that these patients experienced a high death rate (58.4%) and low culture-conversion rates (8.4%) over a follow-up period of 143 days. They concluded that only 1.7 drugs per patient could be regarded as “effective” on the basis of DST results, previous treatment records, or both. Given that this study was conducted concurrently with ours, it is highly likely that a large proportion of their patients were also infected with XDR TB strains with an atypical Beijing genotype. Thus, the poor treatment outcome may be related to the extent of drug-resistance; however, we cannot exclude the possibility that the atypical Beijing genotype contributes to illness and death. A further concern is the knowledge that this clone is now spreading to other provinces in South Africa, possibly due to migration. In Western Cape Province, an estimated 55% of XDR TB case-patients harbor isolates with the atypical Beijing genotype (*32*).

We acknowledge that this study has several limitations. First, clinical data were not available for this study, and thus it was not possible to establish the effects of drug resistance on treatment outcome. However, we do not believe that the strains reported by Kvasnovsky et al. (*12*) differ from those analyzed in this study because the studies were conducted concurrently. Second, our analysis of a convenience sample may have led to an overestimation of the proportion of pre-XDR TB and XDR TB cases in Eastern Cape Province. Third, our use of mutational data to categorize patient isolates as MDR ss, pre-XDR, and XDR is not the accepted standard. However, genetic DST has been endorsed by WHO for first-line anti-TB drugs, and mounting evidence indicates that high confidence mutations accurately predict second-line drug resistance (*33*).

The diagnostic dilemma facing TB control managers in Eastern Cape Province is how to rapidly identify case-patients at risk of harboring the atypical Beijing genotype to prioritize DST, ensure patient isolation, and administer appropriate treatment. Previous studies have shown a strong association between *inhA* promoter mutations and pre-XDR TB and XDR TB (*34*). Given that the Genotype MTBDR*plus* test (*35*) has been implemented as the diagnostic standard in most NHLS laboratories in South Africa, we propose that this test could be used as a rapid screening tool to identify patients harboring drug-resistant atypical Beijing strains (*34*). To contain the spread of this virtually untreatable form of TB, control managers must make use of this information.

Technical Appendix Table showing the geographic distribution of atypical Beijing genotype Mycobacterium tuberculosis isolates and their mutation patterns, and 2 figures showing IS6110 DNA fingerprint patterns of a subset of atypical Beijing pre–extensively drug-resistant tuberculosis isolates and of a subset of atypical Beijing extensively drug-resistant tuberculosis isolates and their geographic origin, South Africa, 2008–2009.

## References

[1] World Health Organization, WHO-IUTALD Global Project on Anti-Tuberculosis Drug Resistance Surveillance. Anti-tuberculosis drug resistance in the world (report no. 4) [cited 2008 Apr 30]. http://www.who.int/tb/publications/2008/drs_report4_26feb08.pdf

[2] Cox HS, McDermid C, Azevedo V, Muller O, Coetzee D, Simpson J, Epidemic levels of drug resistant tuberculosis (MDR and XDR-TB) in a high HIV prevalence setting in Khayelitsha, South Africa. PLoS ONE. 2010;5:e13901 and. 10.1371/journal.pone.0013901 21085569 PMC2981525

[3] World Health Organization. Multidrug and extensively drug-resistant TB (M/XDR-TB) 2010. Global report on surveillance and response [cited 2010 Jun 26]. http://whqlibdoc.who.int/publications/2010/9789241599191_eng.pdf

[4] National Health Laboratory Services. National Institute for Communicable Diseases annual report 2009 [cited 2012 Jan 26]. http://www.nicd.ac.za/assets/files/Annual_report_2009.pdf

[5] Shean KP, Willcox PA, Siwendu SN, Laserson KF, Gross L, Kammerer S, Treatment outcome and follow-up of multidrug-resistant tuberculosis patients, West Coast/Winelands, South Africa, 1992–2002. Int J Tuberc Lung Dis. 2008;12:1182–9 . 18812049

[6] Dheda K, Shean K, Zumla A, Badri M, Streicher EM, Page-Shipp L, Early treatment outcomes and HIV status of patients with extensively drug-resistant tuberculosis in South Africa: a retrospective cohort study. Lancet. 2010;375:1798–807 and. 10.1016/S0140-6736(10)60492-8 20488525

[7] Van Rie A, Warren R, Richardson M, Gie RP, Enarson DA, Beyers N, Classification of drug-resistant tuberculosis in an epidemic area. Lancet. 2000;356:22–5 and. 10.1016/S0140-6736(00)02429-6 10892760

[8] Johnson R, Warren RM, van der Spuy GD, Gey Van Pittius NC, Theron D, Streicher EM, Drug-resistant tuberculosis epidemic in the Western Cape driven by a virulent Beijing genotype strain. Int J Tuberc Lung Dis. 2010;14:119–21 . 20003705

[9] Johnson R, Warren R, Strauss OJ, Jordaan AM, Falmer AA, Beyers N, An outbreak of drug-resistant tuberculosis caused by a Beijing strain in the Western Cape, South Africa. Int J Tuberc Lung Dis. 2006;10:1412–4 . 17167961

[10] Strauss OJ, Warren RM, Jordaan A, Streicher EM, Hanekom M, Falmer AA, Spread of a low-fitness drug-resistant *Mycobacterium tuberculosis* strain in a setting of high human immunodeficiency virus prevalence. J Clin Microbiol. 2008;46:1514–6 and. 10.1128/JCM.01938-07 18272712 PMC2292903

[11] Gagneux S, Long CD, Small PM, Van T, Schoolnik GK, Bohannan BJ. The competitive cost of antibiotic resistance in *Mycobacterium tuberculosis.* Science. 2006;312:1944–6 and. 10.1126/science.1124410 16809538

[12] Kvasnovsky CL, Cegielski JP, Erasmus R, Siwisa NO, Thomas K, der Walt ML. Extensively drug-resistant TB in Eastern Cape, South Africa: high mortality in HIV-negative and HIV-positive patients. J Acquir Immune Defic Syndr. 2011;57:146–52 and. 10.1097/QAI.0b013e31821190a3 21297482

[13] Gandhi NR, Moll A, Sturm AW, Pawinski R, Govender T, Lalloo U, Extensively drug resistant tuberculosis as a cause of death in patients co-infected with tuberculosis and HIV in a rural area of South Africa. Lancet. 2006;368:1575–80 and. 10.1016/S0140-6736(06)69573-1 17084757

[14] Van Rie A, Enarson D. XDR tuberculosis: an indicator of public-health negligence. Lancet. 2006;368:1554–6 and. 10.1016/S0140-6736(06)69575-5 17084741

[15] Sharma M, Thibert L, Chedore P, Shandro C, Jamieson F, Tyrrell G, Canadian multicenter laboratory study for standardized second-line antimicrobial susceptibility testing of *Mycobacterium tuberculosis.* J Clin Microbiol. 2011;49:4112–6 and. 10.1128/JCM.05195-11 21998413 PMC3232997

[16] Warren RM, Victor TC, Streicher EM, Richardson M, Beyers N, van Pittius NC, Patients with active tuberculosis often have different strains in the same sputum specimen. Am J Respi Crit Care Med. 2004;169:610–4. 10.1164/rccm.200305-714OC 14701710

[17] Kamerbeek J, Schouls L, Kolk A, van Agterveld M, van Soolingen D, Kuijper S, Simultaneous detection and strain differentiation of *Mycobacterium tuberculosis* for diagnosis and epidemiology. J Clin Microbiol. 1997;35:907–14 . 9157152 10.1128/jcm.35.4.907-914.1997 PMC229700

[18] Streicher EM, Victor TC. van der SG, Sola C, Rastogi N, van Helden PD, et al. Spoligotype signatures in the *Mycobacterium tuberculosis* complex. J Clin Microbiol. 2007;45:237–40. 10.1128/JCM.01429-06 PMC1828946 17065260

[19] Mokrousov I, Narvskaya O, Otten T, Vyazovaya A, Limeschenko E, Steklova L, Phylogenetic reconstruction within *Mycobacterium tuberculosis* Beijing genotype in northwestern Russia. Res Microbiol. 2002;153:629–37 and. 10.1016/S0923-2508(02)01374-8 12558181

[20] Plikaytis BB, Marden JL, Crawford JT, Woodley CL, Butler WR, Shinnick TM. Multiplex PCR assay specific for the multidrug-resistant strain W of *Mycobacterium tuberculosis.* J Clin Microbiol. 1994;32:1542–6 . 7915723 10.1128/jcm.32.6.1542-1546.1994 PMC264034

[21] Warren R, de Kock M, Engelke E, Myburgh R, Gey van Pittius NC, Victor T, Safe *Mycobacterium tuberculosis* DNA extraction method that does not compromise integrity. J Clin Microbiol. 2006;44:254–6 and. 10.1128/JCM.44.1.254-256.2006 16390984 PMC1351970

[22] Calver AD, Falmer AA, Murray M, Strauss OJ, Streicher EM, Hanekom M, Emergence of increased resistance and extensively drug-resistant tuberculosis despite treatment adherence, South Africa. Emerg Infect Dis. 2010;16:264–71 and. 10.3201/eid1602.090968 20113557 PMC2958014

[23] Sirgel FA, Tait M, Warren RM, Streicher EM, Bottger EC, van Helden PD, Mutations in the *rrs* A1401G gene and phenotypic resistance to amikacin and capreomycin in *Mycobacterium tuberculosis.* Microb Drug Resist. 2012;18:193–7 . 10.1089/mdr.2011.0063 21732736

[24] Mlambo CK, Warren RM, Poswa X, Victor TC, Duse AG, Marais E. Genotypic diversity of extensively drug-resistant tuberculosis (XDR-TB) in South Africa. Int J Tuberc Lung Dis. 2008;12:99–104 . 18173885

[25] Pillay M, Sturm AW. Evolution of the extensively drug-resistant F15/LAM4/KZN strain of *Mycobacterium tuberculosis* in KwaZulu-Natal, South Africa. Clin Infect Dis. 2007;45:1409–14 and. 10.1086/522987 17990220

[26] Porteous JB. The treatment of pulmonary tuberculosis. S Afr Med J. 1959;33:265–7 . 13646880

[27] Ioerger TR, Koo S, No EG, Chen X, Larsen MH, Jacobs WR Jr, Genome analysis of multi- and extensively-drug-resistant tuberculosis from KwaZulu-Natal, South Africa. PLoS ONE. 2009;4:e7778 and. 10.1371/journal.pone.0007778 19890396 PMC2767505

[28] Streicher EM, Muller B, Chihota V, Mlambo C, Tait M, Pillay M, Emergence and treatment of multidrug resistant (MDR) and extensively drug-resistant (XDR) tuberculosis in South Africa. Infect Genet Evol. 2012;12:686–94 and. 10.1016/j.meegid.2011.07.019 21839855

[29] Hillemann D, Rusch-Gerdes S, Richter E. Feasibility of the GenoType MTBDR*sl* assay for fluoroquinolone, amikacin-capreomycin, and ethambutol resistance testing of *Mycobacterium tuberculosis* strains and clinical specimens. J Clin Microbiol. 2009;47:1767–72 and. 10.1128/JCM.00081-09 19386845 PMC2691112

[30] Velayati AA, Masjedi MR, Farnia P, Tabarsi P, Ghanavi J, Ziazarifi AH, Emergence of new forms of totally drug-resistant tuberculosis bacilli: super extensively drug-resistant tuberculosis or totally drug-resistant strains in Iran. Chest. 2009;136:420–5 and. 10.1378/chest.08-2427 19349380

[31] World Health Organization Stop TB Department. Drug-resistant tuberculosis: frequently asked questions, January 26, 2012 [cited 2012 Feb 8]. http://www.who.int/tb/challenges/mdr/TDRFAQs160112final.pdf

[32] Chihota VN, Muller B, Mlambo CK, Pillay M, Tait M, Streicher EM, The population structure of multi- and extensively drug-resistant tuberculosis in South Africa. J Clin Microbiol. 2012;50:995–1002 and. 10.1128/JCM.05832-11 22170931 PMC3295122

[33] Sandgren A, Strong M, Muthukrishnan P, Weiner BK, Church GM, Murray MB. Tuberculosis drug resistance mutation database. 2009. PLoS Med. 2009;6:e2 and. 10.1371/journal.pmed.1000002 19209951 PMC2637921

[34] Müller B, Streicher EM, Hoek KG, Tait M, Trollip A, Bosman ME, *inh*A promoter mutations: a gateway to extensively drug-resistant tuberculosis in South Africa? Int J Tuberc Lung Dis. 2011;15:344–51 . 21333101

[35] Barnard M, Albert H, Coetzee G, O’Brien R, Bosman ME. Rapid molecular screening for multidrug-resistant tuberculosis in a high-volume public health laboratory in South Africa. 2008. Am J Respi Crit Care Med. 2008;177:787–92. 10.1164/rccm.200709-1436OC 18202343

