# Supplementary material for: Emergence and Spread of Extensively and Totally Drug-Resistant Tuberculosis, South Africa
Source: Emerg Infect Dis. 2013 Mar;19(3):449–55. doi: 10.3201//EID1903.120246 (PMC3647643; doi:10.3201//EID1903.120246)
Supplement: Technical Appendix — Table showing the geographic distribution of atypical Beijing genotype Mycobacterium tuberculosis isolates and their mutation patterns, and 2 figures showing IS6110 DNA fingerprint patterns of a subset of atypical Beijing pre–extensively drug-resistant tuberculosis isolates and of a subset of atypical Beijing extensively drug-resistant tuberculosis isolates and their geographic origin, South Africa, 2008–2009. [file 12-0246-Techapp-s1.pdf]

Article DOI: http://dx.doi.org/10.3201/eid1903.120246

Emergence and Spread of Extensively and Totally Drug-Resistant Tuberculosis, South Africa

Technical Appendix

Technical Appendix Table. Geographic distribution of atypical Beijing genotype isolates and their mutation patterns, South Africa, 2008–2009*

Number of isolates harboring a defined mutation in genes known to confer drug resistance

<table>
<tr><th>katG</th><th>rrs</th><th>inhA promoter</th><th>embB</th><th>pncA</th><th>rpoB</th><th>rrs</th><th>gyrA</th><th>MP†</th><th>DR‡</th><th>District municipality</th></tr>
<tr><td>315ACC</td><td>513CAC</td><td>G-17A</td><td>306ATA</td><td>WT</td><td>516GTC</td><td>A1401G</td><td>WT*</td><td rowspan="2">1</td><td rowspan="2">Pre-XDR</td><td rowspan="2">OT</td></tr>
<tr><td rowspan="24">216</td><td rowspan="24">209</td><td rowspan="24">162</td><td rowspan="24">161</td><td>1</td><td>1</td><td>1</td><td>1</td></tr>
<tr><td>Ins172G</td><td>516GTC</td><td>WT</td><td>WT</td><td rowspan="2">2</td><td rowspan="2">MDR</td><td rowspan="2">AM, NMB</td></tr>
<tr><td rowspan="22">159</td><td rowspan="21">149</td><td rowspan="5">6</td><td>3</td></tr>
<tr><td>94GGC</td><td rowspan="2">3</td><td rowspan="2">Pre-XDR</td><td rowspan="2">NMB, CC</td></tr>
<tr><td>2</td></tr>
<tr><td>94GCC</td><td rowspan="2">4</td><td rowspan="2">Pre-XDR</td><td rowspan="2">NMB</td></tr>
<tr><td>1</td></tr>
<tr><td>A1401G</td><td>WT</td><td rowspan="2">5</td><td rowspan="2">Pre-XDR</td><td rowspan="2">AM, NMB, CC, OT</td></tr>
<tr><td rowspan="15">143</td><td>62</td></tr>
<tr><td>94GGC</td><td rowspan="2">6</td><td rowspan="2">XDR§</td><td rowspan="2">AM, NMB, CC, CH</td></tr>
<tr><td>47</td></tr>
<tr><td>94AAC</td><td rowspan="2">7</td><td rowspan="2">XDR</td><td rowspan="2">AM, NMB</td></tr>
<tr><td>10</td></tr>
<tr><td>94CAC</td><td rowspan="2">8</td><td rowspan="2">XDR</td><td rowspan="2">AM, NMB</td></tr>
<tr><td>12</td></tr>
<tr><td>94GCC</td><td rowspan="2">9</td><td rowspan="2">XDR</td><td rowspan="2">NMB</td></tr>
<tr><td>2</td></tr>
<tr><td>94TAC</td><td rowspan="2">10</td><td rowspan="2">XDR</td><td rowspan="2">AM, NMB</td></tr>
<tr><td>4</td></tr>
<tr><td>90GTG</td><td rowspan="2">11</td><td rowspan="2">XDR</td><td rowspan="2">AM</td></tr>
<tr><td>2</td></tr>
<tr><td>Unique mutations</td><td rowspan="2">12–16</td><td rowspan="2">XDR</td><td rowspan="2">AM, NMB</td></tr>
<tr><td>5</td></tr>
<tr><td>516TCC</td><td>WT</td><td>WT</td><td>17</td><td>MDR</td><td>AM, CH</td></tr>
</table>

Number of isolates harboring a defined mutation in genes known to confer drug resistance

katG	*rrs*	*inhA* promoter	*embB*	*pncA*	*rpoB*	*rrs*	*gyrA*	MP†	DR‡	District municipality
					10	4	4			
						A1401G	WT*	18	Pre-XDR	AM, OT
						6	2			
							Unique mutations	19–22	XDR	NMB
							4			
				14CGC	516TCC	WT	WT	23	MDR	AM
				1	1	1	1			
			306ATC	Ins172G	516TCC	A1401G	94GGC	24	XDR	NMB
			1	1	1	1	1			

katG	*rrs*	*inhA* promoter	*embB*	*pncA*	*rpoB*	*rrs*	*gyrA*	MP†	DR‡	District municipality
(315ACC 216)	(513CAC 209)	–15	306ATC	14CGC	531TTG	WT	WT*	25	MDR	AM
		20	17	17	17	1	1			
						A1401G	WT	26	Pre-XDR	AM
						16	5			
							90GTG	27	XDR§	AM, NMB, OT, AN
							11			
			306GTG	31AGC	531TTG	WT	90GTG	28	Pre-XDR	NMB
			3	2	3	2	3			
				WT		A1401G		29	XDR	NMB
				1		1				
		WT	WT	14GCG	531TTG	WT	WT	30	MDR	NMB
		27	1	1	1	1	1			
			306ATA	14CGC	531TTG	WT	WT	31	MDR	NMB
			1	1	1	1	1			
			306ATC	WT	526TAC	WT	WT	32	MDR	NMB
			25	3	3	3	3			
				14CGC	516GTC	WT	88TGC	33	Pre-XDR	CH§§
				22	1	1	1			
					531TTG	WT	WT	34	MDR	AM, NMB, OT, AN
					21	17	12			
							91CCG	35	Pre-XDR	NMB
							3			
							Unique mutations	36–37	Pre-XDR	AM,NMB
							2			
						A1401G	WT	38	Pre-XDR	AM, NMB
						4	2			

							Unique mutations	39–40	XDR	AM, NMB
							2			
	WT	WT	WT	WT	516TAC	WT	WT	41	MDR	NMB
	7	6	4	4	2	1	1			
						A1401G	94GCC	42	XDR	NMB
						1	1			
					MIX	MIX	94GCC	43	XDR	CC
					1	1	1			
					531TTG	WT	WT	44	MDR	NMB
					1	1	1			
			306ATA	34TAG	516TAC	WT	MIX	45	pre-XDR	CC
			2	2	2	1	1			
						A1401G	WT	46	Pre-XDR	AM
						1	1			
		−15	WT	WT	531TTG	A1401G	94GCC	47	XDR	AM
		1	1	1	1	1	1			
WT	WT	−15	WT	NR	531TTG	WT	WT	48	MDR	NMB
1	1	1	1	1	1	1	1			

*MP, mutation pattern; DR, drug resistance; WT, wild type; pre–XDR, pre-extensively drug resistant; OT, OR Tambo; Am, Amathole; NMB, Nelson Mandela Ba7; MDR, multidrug resistant; CC, Cacadu; CH, Chris Hani; An, Alfred Nzo.

Technical Appendix Figure 1. IS*6110* DNA fingerprint patterns of a subset (63/85) of atypical Beijing pre–extensively drug-resistant tuberculosis isolates and their geographic origin, South Africa, 2008–2009.

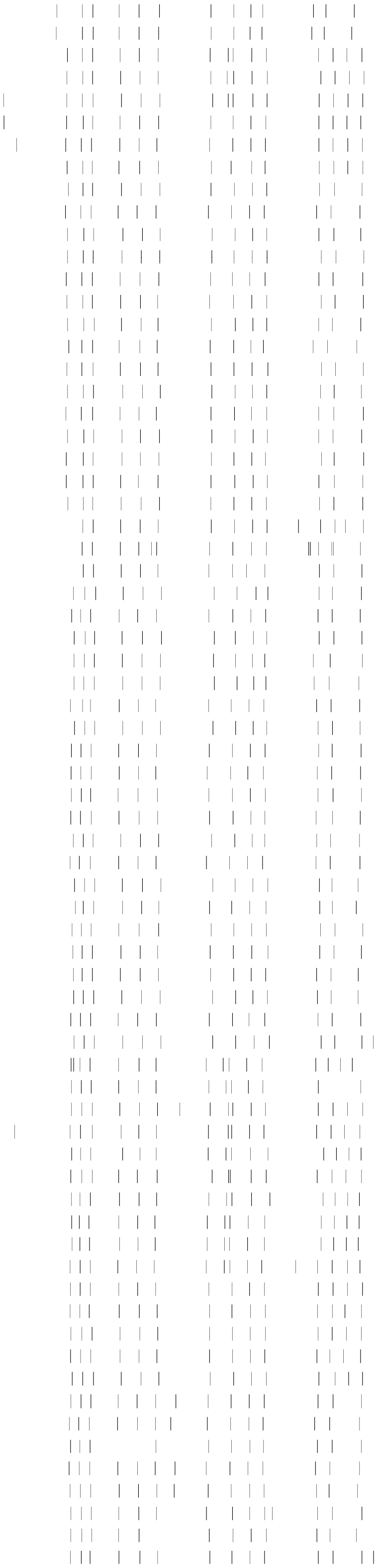

IS*6110* DNA fingerprint pattern	n	Mutation pattern	Cluster	Clinic	District Municipality
	1	MP6	12050	Empilweni	NMB
	1	MP8	12050	Mdantsane	AM
	1	MP6	12051	New Brighton	NMB
	1	MP6	12051	Chatty	NMB
	1	MP9	12052	Mabandla	NMB
	1	MP10	12053	Motherwell	NMB
	1	MP8	12054	Soweto	NMB
	1	MP6	12068	Chatty	NMB
	3	MP8	12055	Jose Pearson	NMB
	1	MP8	12055	Silvertown	NMB
	2	MP8	12055	Schauder	NMB
	1	MP8	12055	Chatty	NMB
	3	MP8	12055	Kwazakhele	NMB
	1	MP8	12055	Fort Grey	AM
	1	MP8	12055	Ezibeleni	AM
	1	MP8	12055	Port Alfred	CC
	1	MP5	12055	St Albans Prison	NMB
	1	MP5	12055	Govan Mbeki	NMB
	1	MP6	12055	Masakhane	NMB
	1	MP6	12055	Tshangana	NMB
	1	MP7	12055	Mdantsane	AM
	1	MP17	12055	Empilweni	NMB
	1	MP5	12029	Empilweni	NMB
	1	MP8	12020	Jose Pearson	NMB
	1	MP47	12021	Nkqubela	AM
	1	MP43	12014	Themba	NMB
	1	MP2	86	Frankfort	AM
	1	MP5	86	Jose Pearson	NMB
	1	MP5	86	Schauder	NMB
	1	MP6	86	Wells Estate	NMB
	1	MP8	86	Motherwell	NMB
	1	MP8	86	Kwazakhele	NMB
	1	MP8	86	Zwide	NMB
	1	MP8	86	Walmer	NMB
	1	MP8	86	Kwamagxaki	NMB
	1	MP8	86	Ndevana	AM
	1	MP8	86	Mdantsane	AM
	1	MP8	86	Glenmore	AM
	1	MP8	86	Nkwenkwezi	CC
	1	MP10	86	Openshaw	AM
	1	MP19	86	Kwazakhele	NMB
	1	MP24	86	Jose Pearson	NMB
	1	MP25	86	Zanempilo, Bisho	NMB
	1	MP25	86	Nu	AM
	1	MP25	86	Nkqubela	AM
	1	MP32	86	Mdantsane	AM
	1	MP8	11973	Lunga Kobese	NMB
	1	MP8	12040	Empilweni	NMB
	1	MP8	12011	Walmer	NMB
	1	MP6	12018	Fort Grey	AM
	1	MP29	12017	Motherwell	NMB
	1	MP5	11846	Frankfort	AM
	1	MP8	11846	Motherwell	NMB
	1	MP8	11846	Wells Estate	NMB
	1	MP8	11846	Jama	AM
	1	MP18	11846	Rosedale	NMB
	1	MP8	12009	Motherwell	NMB
	1	MP8	12043	Nompumelelo	AM
	1	MP8	12043	Lizo Ngcana	NMB
	1	MP8	12043	Motherwell	NMB
	1	MP25	12043	Gompo	AM
	1	MP25	12043	Mt Frere, Trnsk	OT
	1	MP10	12022	Walmer	NMB
	1	MP8	12022	Fort Grey	AM
	1	MP8	12023	Fort Grey	AM
	2	MP10	12028	Walmer	NMB
	1	MP4	11846	Port Elizabeth	NMB
	1	MP1	11845	Ginsberg	AM
	1	MP8	11819	Jose Pearson	NMB
	1	MP8	11838	Kwazakhele	NMB

Technical Appendix Figure 2. IS*6110* DNA fingerprint patterns of a subset (81/103) of atypical Beijing extensively drug-resistant tuberculosis isolates and their geographic origin, South Africa, 2008–2009

IS*6110* DNA fingerprint pattern

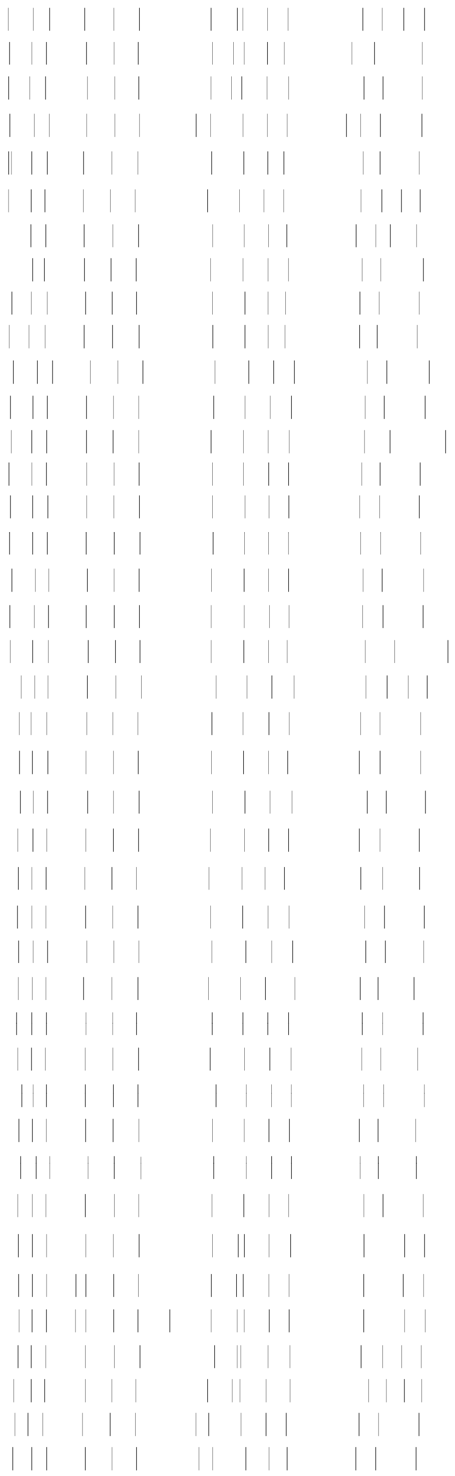

n	Mutation pattern	Cluster	Clinic	District Municipality
1	MP11	11846	Empilweni	NMB
1	MP11	11822	Uitenhage	NMB
1	MP11	11822	Chatty	NMB
1	MP11	11848	Winterberg	AM
1	MP11	12041	Nkqubela	AM
1	MP11	12057	Alicedale	CC
1	MP11	11252	Dimbaza	AM
1	MP11	12030	Booyens Park	NMB
1	MP11	12055	Helenvale	NMB
1	MP11	12055	West End	NMB
2	MP11	12055	Zwide	NMB
1	MP11	12055	Kwamagxaki	NMB
1	MP11	12055	Tanduxolo	NMB
1	MP11	12055	Kirkwood	CC
1	MP11	12055	Temba	CC
1	MP11	12055	Marselle	CC
1	MP11	12055	Canzibe	OT
1	MP11	12055	Nolita	OT
1	MP11	12065	Temba	CC
1	MP11	12061	Isolomzi	NMB
1	MP11	86	Tanduxolo	NMB
2	MP11	86	Booyens Park	NMB
1	MP11	86	New Brighton	NMB
1	MP11	86	Uitkyk	NMB
2	MP11	86	Walmer	NMB
2	MP11	86	Empilweni	NMB
1	MP11	86	Motherwell	NMB
1	MP11	86	Govan Mbeki	NMB
1	MP11	86	Soweto, PE	NMB
1	MP11	86	Nkqubela	AM
1	MP11	86	Sweetwaters	AM
1	MP11	86	Bezville	AM
1	MP11	86	Temba	CC
1	MP11	86	Korsten, PE	CC
1	MP11	12060	Motherwell	NMB
1	MP11	12024	Zwide	NMB
1	MP11	12063	Max Madlingozi	NMB
1	MP11	11846	Tshabo	AM
1	MP11	11846	Mabandla	NMB
1	MP11	11840	Max Madlingozi	NMB
1	MP11	11840	Walmer	NMB
